# Circulating IL-17 Level Is Positively Associated with Disease Activity in Patients with Systemic Lupus Erythematosus: A Systematic Review and Meta-Analysis

**DOI:** 10.1155/2021/9952463

**Published:** 2021-07-21

**Authors:** Rulan Yin, Rong Xu, Lei Ding, Wenjie Sui, Mei'e Niu, Mingjun Wang, Lan Xu, Haifang Wang, Chomphoonut Srirat

**Affiliations:** ^1^Department of Rheumatology, The First Affiliated Hospital of Soochow University, Suzhou, China; ^2^Faculty of Nursing, Chiang Mai University, Chiangmai, Thailand; ^3^Department of Internal Medicine, Suzhou Municipal Hospital, Suzhou, China; ^4^Department of Nursing, The First Affiliated Hospital of Soochow University, Suzhou, China

## Abstract

Previous studies on the relationship between the circulating level of interleukin-17 (IL-17) and disease activity in systemic lupus erythematosus (SLE) were contradictory. This study is aimed at quantitatively assessing the correlation between the circulating IL-17 level and disease activity in SLE patients. A systematic search for related literature was conducted via PubMed, Web of Science, EMBASE, and Cochrane Library (up to January 26, 2021). The relationship between circulating IL-17 levels and SLE activity was evaluated using Fisher's *z* value, which was then converted to *r*. The standardized mean difference (SMD) and its 95% confidence interval (CI) were used to describe the difference between the circulating IL-17 level in patients with active and inactive SLE. STATA 16.0 was used to perform statistical analysis. Random-effects model was performed to synthesize data. Twenty-six studies involving 1,560 SLE patients were included in this review. The pooled *r* value was 0.38 (95% CI: 0.25-0.50; *I*^2^ = 83.8%, *P* < 0.001) between the SLE activity and circulating level of IL-17. Patients with active SLE had higher level of circulating IL-17 than that of inactive (pooled SMD = 0.95, 95% CI: 0.38-1.53; *I*^2^ = 90.5%, *P* < 0.001). The subgroup analysis suggested that the region and detection method of circulating IL-17 might not be a source of heterogeneity. No significant publication bias was found. In summary, circulating IL-17 level has a low positive relationship with SLE activity. It is necessary to carefully consider the use of circulating IL-17 as a biomarker of the disease activity in SLE patients. The relationship between the circulating level of IL-17 and SLE activity should be further confirmed in randomized controlled studies.

## 1. Introduction

Systemic lupus erythematosus (SLE) is a chronic, multifactorial inflammatory autoimmune disease with overproduction of autoantibodies and immune complex (IC) deposition in multiple organs [1]. Although the pathogenesis of SLE remains elusive, from the point of view of the etiology of the occurrence and development of SLE on the molecular level, it can be seen that the imbalance of the immune regulation mechanism plays a key role, especially the imbalance of proinflammatory and anti-inflammatory cytokines [2].

Interleukin-17 (IL-17), a proinflammatory cytokine, is secreted mainly by activated T helper-17 (Th-17) cells, double-negative (DN) T cells, macrophages, and neutrophils [3]. The IL-17 cytokine family composes of six members: IL-17A to IL-17F [4], among which, IL-17A (also known as IL-17) was the first reported cytokine and had been Intensive studied [5, 6]. Therefore, this review will focus on IL-17. A meta-analysis, which included twenty articles published until 22 November 2018, showed that SLE patients had higher circulating IL-17 levels than healthy controls [7], suggesting its possible role in the pathogenesis and disease activity of SLE. However, till now, the relationship between circulating IL-17 levels and SLE activity is still controversial. Elewa et al. [8], Chen et al. [9], and Dedong et al. [10] found a positive relationship between IL-17 levels and activity of SLE, while the positive correlation was not observed in the studies by Abo-Shanab et al. [11], Cavalcanti et al. [12], and Yao et al. [13]. In addition, Yin et al. [14], Mohammadi et al. [15], and Mok et al. [16] found that IL-17 was negatively associated with SLE activity, although the correlation is not significant. As far as we know, no meta-analysis on the relationship between these two variables has been published. In view of this situation, we conducted a systematic review and meta-analysis to gather the available evidence to more accurately evaluate the correlation between the level of circulating IL-17 and disease activity in patients with SLE, so as to provide a recommendation on whether using the circulating IL-17 level as a biomarker of SLE activity.

## 2. Materials and Methods

### 2.1. Search Strategy

This systematic review and meta-analysis was carried out according to the Preferred Reporting Items for Systemic Review and Meta-Analyses (PRISMA) guidelines [17]. A systematic search was performed in the four English databases: PubMed, Web of Science, EMBASE, and Cochrane Library (up to January 26, 2021), with the combined search terms displayed as following: IL-17 (interleukin-17 or interleukin 17 or IL-17 OR IL 17), SLE (lupus or SLE or systemic lupus erythematosus), and disease activity. In addition, the list of references in the included articles were searched to obtain additional studies.

### 2.2. Inclusion Criteria

Inclusion criteria were (all must be met): (1) investigating the relationship between SLE and circulating IL-17 levels; (2) cross-sectional, case-control, or longitudinal design (using baseline data) in humans; (3) reporting data on the correlation between the circulating level of IL-17 and SLE activity, including Spearman's or Pearson's correlation coefficient (*r*) and/or mean ± standard deviation (SD)/median (interquartile range, IQR)/median (range)/mean (range) of the IL-17 circulating level for both active and inactive SLE groups; and (4) published in English.

### 2.3. Exclusion Criteria

Studies were excluded if they were: (1) reviews, meta-analysis, meeting or conference abstracts, case reports, comments, and letters; (2) no full-text studies; (3) duplicated articles and repetitive data (when there are different articles using the same sample from the same unit, select the most recently published one); (4) nonhuman investigation; and (5) ambiguous data description.

### 2.4. Data Extraction and Quality Assessment

Two authors independently screened the literature by reading the title and abstract, as well as further full-text review. After confirming the included studies, the two authors independently extracted data from each paper, including the first author, year of publication, region, sample size and percent of females of active/inactive and total SLE patients, age, disease duration, the mean ± SD/median (IQR)/median (range)/mean (range) of circulating IL-17 level of total sample and / or two groups (active SLE group and inactive SLE group) (pg/ml), detection method of circulating IL-17, definition of active SLE, and Pearson's/Spearman's correlation coefficient (*r*) between SLE activity and the level of circulating IL-17. A modified version of the Newcastle-Ottawa Scale (M-NOS) [18] was used for quality assessment along with data extraction, and scores of ≥3 and <3 were judged as low and high risk of bias, respectively. Any disagreements between two authors were solved through discussion and adjudication by the third author.

### 2.5. Statistical Analysis

STATA 16.0 was used to perform the meta-analysis. The random-effects model was used to synthesize *r* and continuous variables as it is more desirable than fixed-effects model and can provide wider confidence interval (CI). For correlation coefficients (*r*), Spearman's *r* was first converted to Pearson's *r* [19]. Then, the pooled estimate of Pearson's *r* by Fisher's exact test *r*-to-*z*transformation was calculated [20]. All values were weighted by the reciprocal of the *r* variance, after which the combined *r* of the overall value was converted back for presentation. For continuous data in active and inactive SLE groups, the median (IQR) and median (range) of circulating IL-17 was first transformed into mean ± SD [21, 22] and mean (range) into mean ± SD [23]. Then, standardized mean difference (SMD) and its 95% CI were used to synthesize the circulating IL-17 level in the two groups. *I*^2^ was used to evaluate the heterogeneity of cross-studies, and *I*^2^ more than 50% indicated significant heterogeneity. Subgroup and sensitivity analyses were used to search for sources of heterogeneity. Funnel plots and Egger's test were only combined to assess publication bias when ≥10studies were included [24, 25], as the power of these tests is too low to distinguish chance from real asymmetry when there are less than 10 studies [26].

## 3. Results

### 3.1. Study Selection

**Figure 1:**
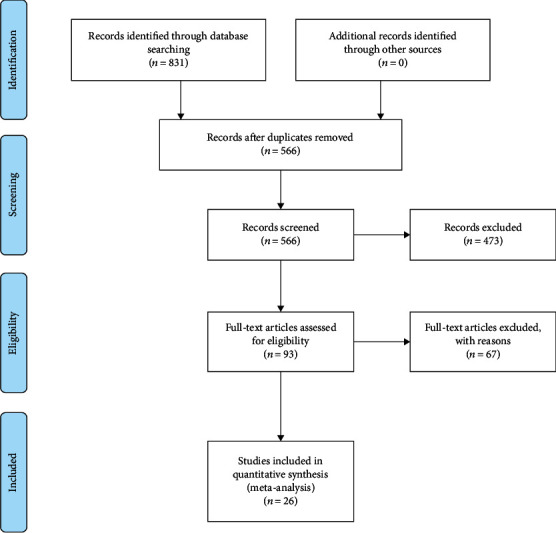
Flowchart of article selection.

After assessing by selection criteria,26 studies were incorporated, which involved 1,560 SLE patients" to "26 studies, which involved 1,560 SLE patients, were incorporated.  Figure 1 showed the flow chart of the study selection process.

### 3.2. Study Characteristics

Table 1 summarized the features of the incorporated literatures. Among the included 26 researches, 10 occurred in China [10, 13, 14, 16, 27–32], 6 in Egypt [11, 33–37], 2 in Brazil [12, 38], 2 in Iran [15, 39], and 1 in each of the following countries: Indonesia [40], India [41], Malaysia [42], Norway [43], Poland [44], and Mexico [45]. ELISA was most commonly to measure circulating IL-17 (23 articles), followed by Cytometric Bead Array Human Th1/Th2/Th17 Cytokine Kit (1 article), Invitrogeńs Novex human Th1/Th2/Th17 Magnetic 10-Plex Panel assay (1 article), and MILLIPLEX MAP human cytokine detection kit (1 article). After assessing by M-NOS (0-5 scores), 4 articles were judged as low risk of bias (≥3 points); the other 22 were high risk of bias (<3 points) (Supplementary 1).

### 3.3. Meta-Analysis of the Correlation between Circulating IL-17 Level and SLE Activity

**Figure 2:**
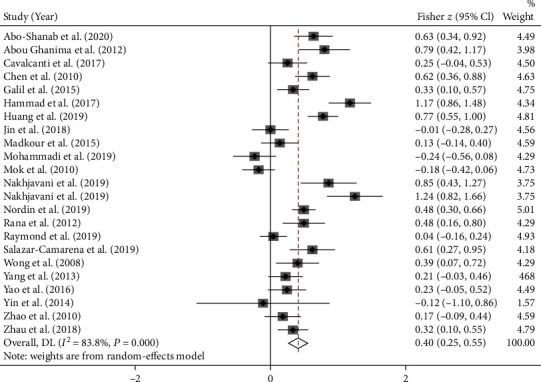
Meta-analysis of the correlation between circulating IL-17 level and SLE activity.

Figure 2 revealed that 22 studies reported the correlation (*r*) between the disease activity and circulating IL-17 level in patients with SLE (Nakhjavani et al. [39] provided two separate *r* between IL-17 level and disease activity in SLE patients with/without lupus nephritis), and the pooled Fisher *z* value was 0.40 (95% CI: 0.25-0.55; *I*^2^ = 83.8%, *P* < 0.001). After *z*-to-*r* back transformation, the pooled *r* was 0.38 (95% CI: 0.25-0.50, *P* < 0.001), suggesting a low positive relationship between the level of circulating IL-17 and SLE activity.

### 3.4. Meta-Analysis Comparing the Circulating IL-17 Levels in Active and Inactive SLE Patients

**Figure 3:**
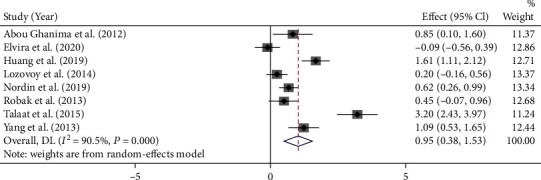
Meta-analysis comparing the circulating IL-17 levels in active and inactive SLE patients.

A total of eight studies compared the level of circulating IL-17 between patients with active and inactive SLE. The result showed that patients with active SLE had higher level of circulating IL-17 than inactive SLE patients (pooled SMD = 0.95, 95% CI: 0.38-1.53; *I*^2^ = 90.5%, *P* < 0.001) (Figure 3).

### 3.5. Subgroup Analysis

**Figure 4:**
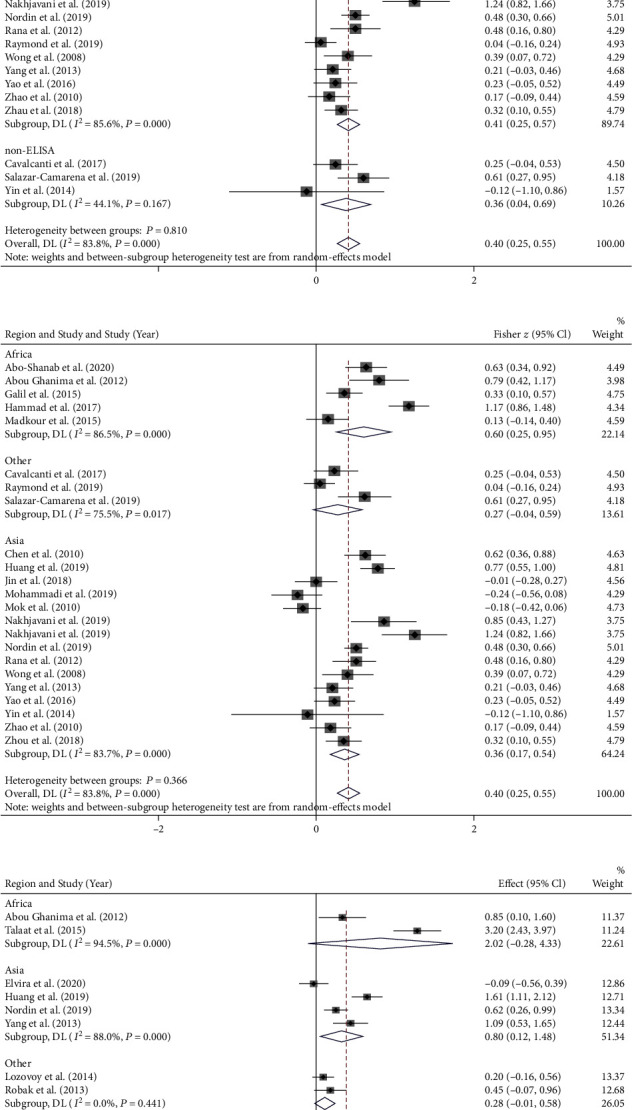
Subgroup analysis of (a) detection method of circulating IL-17 and (b) region in pooled *r* analysis and (c) region in pooled SMD analysis.

The subgroup analysis was performed on the basis of geographic region (Africa, Asia, other) and detection method of circulating IL-17. The results showed that none of the variables might be the source of heterogeneity (Figure 4).

### 3.6. Sensitivity Analysis and Publication Bias

Sensitivity analysis displayed that the results were unchanged when each study was excluded serially (Supplementary 2). For all comparisons between active SLE patients and inactive SLE patients, there is no significant difference if any study was omitted, indicating that the results of this meta-analysis were stable. Egger's test was used to assess the asymmetry of the funnel plot in the pooled *r* analysis, and the result showed that no significant evidence of publication bias was found (Egger bias = 2.17, 95% CI: (-2.05, 6.40), *P* = 0.296) (Supplementary 3). For the meta-analysis comparing the IL-17 level between the active and inactive SLE groups, funnel plot and Egger's test were not conducted as the number of included studies was 8, which was less than 10.

## 4. Discussion

As far as we know, this systematic review and meta-analysis, which included 26 articles involving 1,560 SLE patients, is the first quantitative assessment of the association between circulating level of IL-17 and disease activity in SLE patients. This study indicated that circulating level IL-17 was positively associated with SLE activity, with a low correlation. In addition, active SLE patients had higher level of circulating IL-17 than inactive SLE patients.

The subgroup analysis based on region and circulating IL-17 assay suggested that neither of these variables might be the source of heterogeneity. However, the results showed that the correlation between the circulating IL-17 level and SLE activity was slightly stronger when circulating IL-17 was measured by ELISA (Fisher *z* = 0.41; *Z* = 5.029, *P* < 0.001) than by non-ELISA (Fisher *z* = 0.40; *Z* = 2.200, *P* = 0.028), although no statistically significant difference was found between groups (*P* = 0.810). This may due to the sensitivity of immunosuppressive drugs to ELISA, which is a rapid test and more commonly used because both direct and indirect assay methods can be performed and are highly reactive. Future researchers are recommended to conduct randomized controlled trials to further explore the differences between ELISA and other methods of measuring circulating IL-17. Moreover, the association between SLE activity and circulating level of IL-17 was highest in Africa (Fisher *z* = 0.60; *Z* = 3.346, *P* = 0.001), followed by Asia (Fisher *z* = 0.36; *Z* = 3.824, *P* < 0.001); other regions were not correlated (*Z* = 1.706, *P* = 0.088), and the difference between groups was not statistically significant (*P* = 0.366). It may be due to the differences in economic conditions. Africa is the least developed regions with poor sanitary conditions. Africans usually will not go for hospital treatment until the disease is serious, as they have to reduce the treatment cost. In this case, it makes sense that patients with SLE in Africa who seek medical attention are typically of high disease activity and high levels of circulating IL-17, which is involved in the body's inflammatory response. Compared to Africa, Asia in general has better economic conditions and better health facilities, SLE patients in Asia can see the doctor regularly, rather than waiting for the disease to become severe. However, some patients would seek medical treatment only when the disease was serious, which might explain why the level of circulating IL-17 was significantly higher in active SLE patients than in inactive SLE patients only in Asia (Fisher *z* = 0.80; *Z* = 2.312, *P* = 0.021), not in Africa (*Z* = 1.724, *P* = 0.085). Thus, patients with SLE may have relatively low disease activity when seeking medical treatment, thus forming a low relationship between IL-17 and SLE activity. Notably, further researches need to be conducted to verify this hypothesis.

Previous evidence suggests that both innate and immune systems are involved in SLE pathogenesis [46]. Plasmacytoid dendritic cells (pDC) are activated by apoptotic debris and release type I interferon (IFNs), particularly IFN-*α*. This is conducive to the production of cytokines through DC, which promotes the differentiation of Th-17 cells at the expense of Treg cells. Th-17 cells and DN T cells secrete IL-17 that takes part in the formation of spontaneous germinal centers [46]. IL-17 induces the production of other proinflammatory cytokines, such as IL-1 and IL-6 [47]; cooperates with IL-23 and other Th-17-related cytokines, for instance, IL-17F and IL-21, to form a complex network to promote inflammation response and amplify the tissue damage induced by SLE [48]; and improves B-cell proliferation and excessive production of autoantibodies in SLE patients [49, 50]. The result is increased production of autoantibodies, deposition of immune complexes in the target organs, complement activation, and tissue damage. Eventually, these immune complexes activate pDC [46]. Moreover, the genetic deletion of IL-17 was shown to ameliorate the pathology of SLE [51]. This is the further proof that IL-17 induces the recruitment of immune cells to target tissues, promoting and maintaining the inflammatory process [52], which worsens SLE activity.

The current meta-analysis indicated that the circulating IL-17 level was positively related to SLE activity, while IL-17 being the part of SLE pathogenetic core process. Therefore, the circulating IL-17 level may be clinically important for SLE treatment. Our findings indicated that balancing circulating IL-17 might be a promising treatment target to improve SLE outcome. Actually, two anti-IL-17 therapies have been approved by the U.S. Food and Drug Administration [53]. However, it is important to note that the degree of correlation between the disease activity and circulating level of IL-17 in SLE patients was low (pooled *r* = 0.38). It is necessary to carefully consider the use of circulating IL-17 as a biomarker of disease activity in patients with SLE, and the relationship should be further confirmed in randomized controlled studies.

The strength of this systematic review and meta-analysis was that the inconsistent results of previous studies on the relationship between SLE activity and circulating IL-17 level were quantitatively synthesized, showing a low positive correlation. Since a total of 1,249 patients were included, the sample size was large and the results were stable; thus, the pooled *r* value can provide reference for the treatment of SLE in the future. Moreover, this meta-analysis showed that the circulating IL-17 level was higher in patients with active SLE (*n* = 300) than those with inactive SLE (*n* = 306). This may be helpful to set up the cut-off of circulating IL-17 in SLE in the future and help determine the disease activity of SLE patients through the circulating level of IL-17.

However, there are several limitations to this meta-analysis. First, most of the included studies were conducted in Africa and Asia, while the remaining three were carried out in South America, North America, and Europe. There were no studies from Oceania due to some unknown reason, which limited the representativeness of the results to a certain extent. We recommend that relevant studies be conducted in these two continents and that the results of this meta-analysis be updated to better represent the global level. Second, different designs of the included studies may lead to heterogeneity among literature. Third, despite the use of a random-effects model, as well as subgroup analysis and sensitivity analysis, significant heterogeneity still existed in the pooled analysis that could not be explained. Fourth, we did not compare circulating levels of IL-17 between patients with SLE and health controls, as the result were recently published. Last but not least, several articles that met other inclusion criteria but had ambiguous data description were excluded, which probably affected the reliability of the pooled results. Therefore, we hope researchers can clearly describe the data in future articles, so as to avoid other researchers excluding the literature that should have been included in the analysis due to ambiguous data description when summarizing evidence.

## 5. Conclusions

Circulating levels of IL-17 have a low positive relationship with SLE activity, and patients with active SLE have higher circulating IL-17 level than inactive SLE patients. Given this low correlation, rheumatologists need to be cautious when considering circulating IL-17 as a biomarker and targeting IL-17 therapy in SLE patients. Before this, further rigorous randomized controlled trials are needed to confirm the relationship between the level of circulating IL-17 and disease activity in patients with SLE.

## Figures and Tables

**Table 1:** Characteristics of the incorporated studies.

Study	Country	Sample size (Ac/In-ac)	Female (%) Ac/In-ac	Age (y) Ac/In-ac	Disease duration (y) Ac/In-ac	IL-17 (pg/ml) Ac/In-ac	Detection methods	Definition of active SLE	*r*	Quality
Abo-Shanab et al. (2020)	Egypt	50	100	24 ± 6.7	11.13 ± 5.01	36.74 ± 39.42	ELISA	NA	0.558	2
Abou Ghanima et al. (2012)	Egypt	30 (15/15)	93.3/86.7	33 ± 7/34 ± 8	9.1 ± 2.3/8.6 ± 3.6	25.96 ± 4.84/20.44 ± 7.78	ELISA	SLEDAI ≥ 6	0.661^∗∗^	3
Cavalcanti et al. (2017)	Brasil	51 (26/25)	92	14.57 ± 3.33	3.35 ± 1.78	4.93 ± 10.10	Cytometric Bead Array Human Th1/Th2/Th17 Cytokine kit	SLEDAI‐2 k ≥ 4	0.242	3
Chen et al. (2010)	China	60	100	18-49^$^	NA	19.67 ± 5.76	ELISA	SLEDAI>12 (severe)	0.549^∗∗^	2
Elvira et al. (2020)	Indonesia	68 (34/34)	100	32.52 ± 9.96	17.6% (≤1 yr)	19.67 ± 1.299/19.78 ± 1.187	ELISA	MEX‐SLEDAI > 2	NA	2
Galil et al. (2015)	Egypt	72 (30/42)	100	34.23 ± 8.15	4.62 ± 2.73	19.47 ± 10.21	ELISA	SLEDAI-2 K ≥4 and have active lupus nephritis	0.322^∗∗^	2
Hammad et al. (2017)	Egypt	42 (42/0)	81.0/0	14.28 ± 3.84	2.50 ± 3.52	24.46 ± 7.87	ELISA	NA	0.824^∗∗∗^	2
Huang et al. (2019)	China	80 (37/43)	70.3/67.4	43.3 ± 11.3/39.9 ± 9.1	NA	9.08 ± 1.39/6.82 ± 1.41	ELISA	SLEDAI > 9	0.648^∗∗∗^	2
Jin et al. (2018)	China	55	94.6	37.7 ± 13.6	4.50 ± 6.80	4.57 ± 1.21	ELISA	SLEDAI‐2 K > 4 (more active)	-0.005	2
Lozovoy et al. (2014)	Brazil	123 (53/70)	90.6/94.3	41.41 ± 15.62/41.09 ± 16.27	NA	5.26 ± 4.53/4.41 ± 3.97	NA	Decreased C3 (<90 mg/dL) and/or decreased C4 (<10 mg/dL) and/or positive anti-double-stranded DNA (anti-dsDNA; titre 1/10).	NA	3
Madkour et al. (2015)	Egypt	57	94.7	29.9 ± 8.2	7.4 ± 5.3	27.8 ± 11.6	ELISA	NA	0.130	1
Mohammadi et al. (2019)	Iran	40	100	NA	NA	NA	ELISA	NA	-0.238	1
Mok et al. (2010)	China	70 (36/34)	92.9	45.2 ± 12.0	12.9 ± 8.0	14.8/14.8^#^	ELISA	SLEDAI > 6	-0.178	2
Nakhjavani et al. (2019)	Iran	50 (44/6)	80	36.7 ± 11	NA	24.05 ± 4.81	ELISA	SLEDAI ≥ 6	0.692^∗∗∗^ (*n* = 25-no LN) 0.845^∗∗∗^ (*n* = 25-LN)	2
Nordin et al. (2019)	Malaysia	120 (56/64)	89.2	41.9 ± 12.5	11.8 ± 7.2	42.88 ± 13.05/35.99 ± 8.99	ELISA	Modified SLEDAI-2 K ≥1	0.447^∗∗∗^	2
Rana et al. (2012)	India	40	85	11.70 ± 2.55	1.35 ± 1.84	766.95 ± 357.82	ELISA	SLEDAI ≥ 10	0.447^∗^	2
Raymond et al. (2019)	Norway	100	87	49^#^	NA	NA	ELISA	SLEDAI‐2 K ≥ 1	0.039	3
Robak et al. (2013)	Poland	60 (28/32)	91.7	39.2 ± 11.25	5.50 ± 5.40	2.89 ± 5.12/1.30 ± 0.89	ELISA	SLEDAI‐2 k ≥ 6	NA	2
Salazar-Camarena et al. (2019)	Mexico	36 (23/13)	100	30.30 ± 9.90/34.54 ± 10.58	4.03 ± 4.26/12.17 ± 6.67	10.46 ± 17.50	Invitrogeńs Novex human Th1/Th2/Th17 Magnetic 10-Plex Panel assay	Mex‐SLEDAI ≥ 3	0.544^∗∗^	2
Talaat et al. (2015)	Egypt	60 (32/28)	93.3	28.58 ± 7.30	4.97 ± 3.41	17.7 ± 2.3/11.4 ± 1.5	ELISA	SLEDAI ≥ 6	NA	2
Wong et al. (2008)	China	40 (3/37)	97.5	38 ± 9	12.8 ± 6.1	26.16 ± 5.82	ELISA	SLEDAI ≥ 6	0.374^∗^	2
Yang et al. (2013)	China	65 (45/20)	91.1/90	34 ± 10/35 ± 12	1.35 ± 1.05/1.80 ± 1.45	88.44 ± 27.91/54.64 ± 37.39	ELISA	SLEDAI≥5	0.211^∗^	2
Yao et al. (2016)	China	50 (36/14)	90	37.5 ± 9.1	7.2 ± 5.8	NA	ELISA	SLEDAI > 4	0.229	2
Yin et al. (2014)	China	79	96.2	34.41 ± 9.41	6.91 ± 6.19	0 ± 0	MILLIPLEX MAP human cytokine detection kit	SLEDAI ≥ 5	-0.117 (*n* = 7)	2
Zhao et al. (2010)	China	57	96.5	35.6 ± 13.0	NA	NA	ELISA	SLEDAI ≥ 10 (more active)	0.173	2
Zhou et al. (2018)	China	77	96.1	35.06 ± 13.60	5.06 ± 6.80	229.25 ± 128.43	ELISA	SLEDAI ≥ 6	0.313^∗∗^	2

^∗^ *P* < 0.05, ^∗∗^*P* < 0.01, and ^∗∗∗^*P* < 0.001. Value presented as number, percentage, mean (^#^), range (^$^), or mean ± standard deviation (SD). Abbreviation: Ac: active; In-ac: inactive; SLE: systemic lupus erythematosus; ELISA: enzyme-linked immunosorbent assay; SLEDAI-2K: systemic lupus erythematosus disease activity index 2000; NA: not applicable; SLEDAI: systemic lupus erythematosus disease activity index; LN: lupus nephritis. Quality was measured by modified Newcastle–Ottawa Scale (M-NOS).

## Data Availability

All data supporting this meta-analysis were from previous studies and datasets cited.

## Supplementary Materials

Supplementary Materials Supplement 1: quality assessment of the included studies measured by M-NOS. Supplementary 2: sensitivity analysis for the pooled results of (A) correlation between circulating IL-17 level and SLE activity and (B) differences between circulating IL-17 level in active and inactive SLE patients. Supplementary 3: publication bias. Figure S1: funnel plot of the pooled r analysis between circulating IL-17 level and SLE activity. Figure S2: Egger's test of the funnel plot in pooled *r* analysis.
