# Supplementary material for: Circulating IL-17 Level Is Positively Associated with Disease Activity in Patients with Systemic Lupus Erythematosus: A Systematic Review and Meta-Analysis
Source: Biomed Res Int. 2021 Jul 21;2021:9952463. doi: 10.1155/2021/9952463 (PMC8318742; doi:10.1155/2021/9952463)
Supplement: Supplementary Materials — Supplement 1: quality assessment of the included studies measured by M-NOS. Supplementary 2: sensitivity analysis for the pooled results of (A) correlation between circulating IL-17 level and SLE activity and (B) differences between circulating IL-17 level in active and inactive SLE patients. Supplementary 3: publication bias. Figure S1: funnel plot of the pooled r analysis between circulating IL-17 level and SLE activity. Figure S2: Egger's test of the funnel plot in pooled r analysis. [file 9952463.f1.zip › Supplementary 1.docx]

**Supplementary 1** Quality assessment of the included studies measured by M-NOS

| **Study ID** | **Representativeness** | **Size** | **Comparability** | **Outcome** | **Statistics** | **Total** |
| --- | --- | --- | --- | --- | --- | --- |
| Abo-Shanab et al (2020) | 0 | 0 | 1 | 0 | 1 | 2 |
| Abou Ghanima et al (2012) | 1 | 0 | 0 | 1 | 1 | 3 |
| Cavalcanti et al (2017) | 1 | 0 | 0 | 1 | 1 | 3 |
| Chen et al (2010) | 0 | 0 | 0 | 1 | 1 | 2 |
| Elvira et al (2020) | 0 | 0 | 0 | 1 | 1 | 2 |
| Galil et al (2015) | 0 | 0 | 0 | 1 | 1 | 2 |
| Hammad et al (2017) | 0 | 0 | 0 | 1 | 1 | 2 |
| Huang et al (2019) | 0 | 0 | 0 | 1 | 1 | 2 |
| Jin et al (2018) | 0 | 0 | 0 | 1 | 1 | 2 |
| Lozovoy et al (2014) | 1 | 0 | 0 | 1 | 1 | 3 |
| Madkour et al (2015) | 0 | 0 | 0 | 0 | 1 | 1 |
| Mohammadi et al (2019) | 0 | 0 | 0 | 0 | 1 | 1 |
| Mok et al (2010) | 0 | 0 | 0 | 1 | 1 | 2 |
| Nakhjavani et al (2019) | 0 | 0 | 0 | 1 | 1 | 2 |
| Nordin et al (2019) | 0 | 0 | 0 | 1 | 1 | 2 |
| Rana et al (2012) | 0 | 0 | 0 | 1 | 1 | 2 |
| Raymond et al (2019) | 1 | 0 | 0 | 1 | 1 | 3 |
| Robak et al (2013) | 0 | 0 | 0 | 1 | 1 | 2 |
| Salazar-Camarena et al (2019) | 0 | 0 | 0 | 1 | 1 | 2 |
| Talaat et al (2015) | 0 | 0 | 0 | 1 | 1 | 2 |
| Wong et al (2008) | 0 | 0 | 0 | 1 | 1 | 2 |
| Yang et al (2013) | 0 | 0 | 0 | 1 | 1 | 2 |
| Yao et al (2016) | 0 | 0 | 0 | 1 | 1 | 2 |
| Yin et al (2014) | 0 | 0 | 0 | 1 | 1 | 2 |
| Zhao et al (2010) | 0 | 0 | 0 | 1 | 1 | 2 |
| Zhou et al (2018) | 0 | 0 | 0 | 1 | 1 | 2 |
| Note: low risk of bias (≥3 points), high risk of bias (<3 points).  Abbreviation: M-NOS: modified Newcastle–Ottawa Scale. | | | | | | |
