# Supplementary material for: Circulating IL-17 Level Is Positively Associated with Disease Activity in Patients with Systemic Lupus Erythematosus: A Systematic Review and Meta-Analysis
Source: Biomed Res Int. 2021 Jul 21;2021:9952463. doi: 10.1155/2021/9952463 (PMC8318742; doi:10.1155/2021/9952463)
Supplement: Supplementary Materials — Supplement 1: quality assessment of the included studies measured by M-NOS. Supplementary 2: sensitivity analysis for the pooled results of (A) correlation between circulating IL-17 level and SLE activity and (B) differences between circulating IL-17 level in active and inactive SLE patients. Supplementary 3: publication bias. Figure S1: funnel plot of the pooled r analysis between circulating IL-17 level and SLE activity. Figure S2: Egger's test of the funnel plot in pooled r analysis. [file 9952463.f1.zip › Supplementary 3.docx]

**Supplementary 3** Publication bias


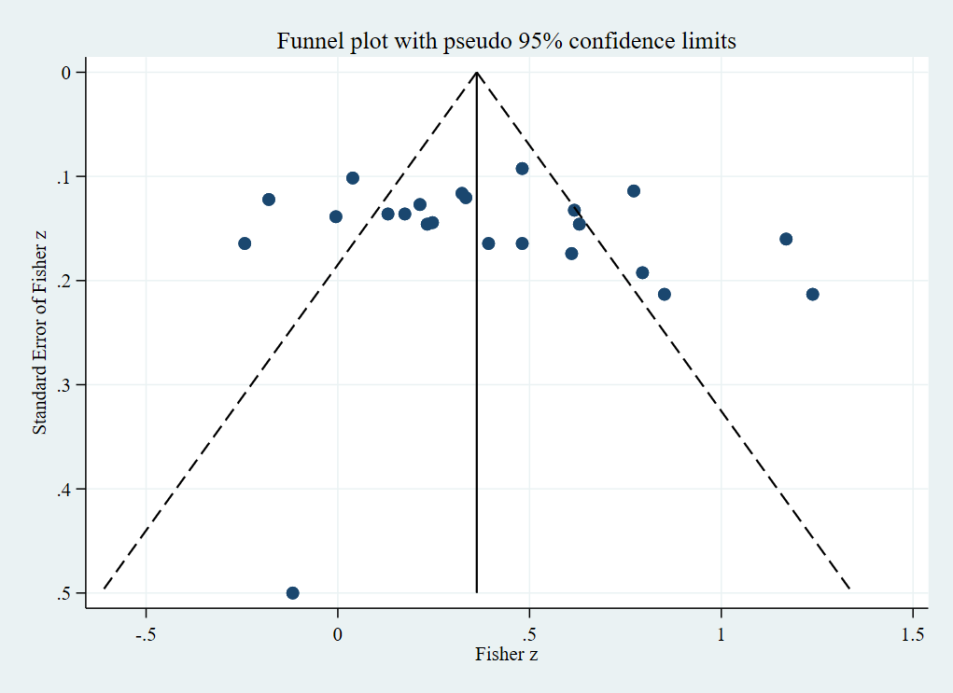


**Figure S1** Funnel plot of the pooled r analysis between circulating IL-17 level and SLE activity


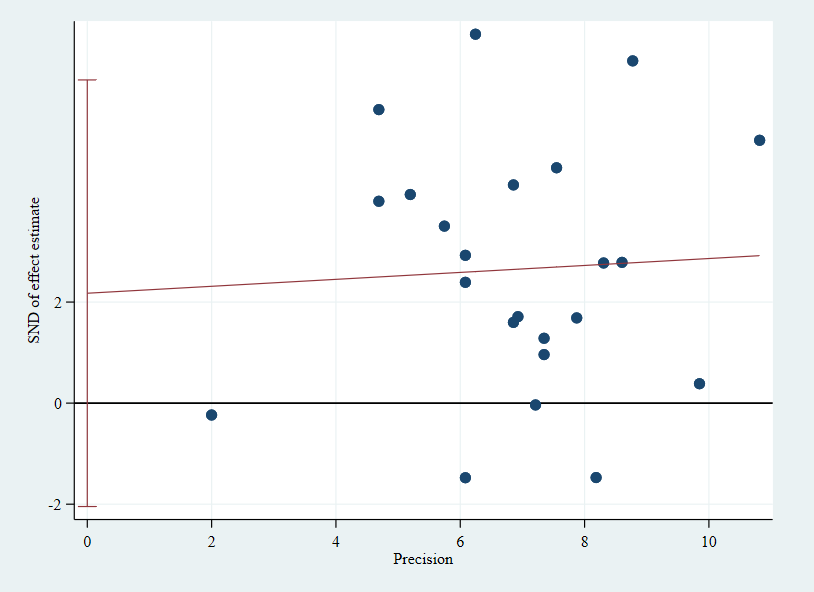


**Figure S2** Egger’s test of the funnel plot in pooled r analysis
